# In Vitro Evaluation of Acrylic Adhesives in Lymphatic Fluids-Influence of Glue Type and Procedural Parameters

**DOI:** 10.3390/biomedicines10051195

**Published:** 2022-05-21

**Authors:** Daniel Kuetting, Patrick Kupczyk, Tatjana Dell, Julian A. Luetkens, Carsten Meyer, Ulrike I. Attenberger, Claus C. Pieper

**Affiliations:** Department of Diagnostic and Interventional Radiology, University of Bonn, Venusberg-Campus 1, 53105 Bonn, Germany; daniel.kuetting@ukbonn.de (D.K.); patrick.kupczyk@ukbonn.de (P.K.); tatjana.dell@ukbonn.de (T.D.); julian.luetkens@ukbonn.de (J.A.L.); carsten.meyer@ukbonn.de (C.M.); ulrike.attenberger@ukbonn.de (U.I.A.)

**Keywords:** acrylic adhesives, lymphatic embolization, NBCA, metacryloxysulpholane

## Abstract

To evaluate the embolic properties of different acrylic adhesive/iodized oil mixtures for lymphatic interventions. Polymerization of histoacryl (HA) (Bayer Healthcare) and glubran 2 (GL) (GEM) mixed with iodized oil (ratios 1:0–1:7) were investigated in lymphatic fluids with low and high triglyceride (low TG & high TG) contents. Static polymerization time and dynamic polymerization experiments with different volumes of glucose flush (1, 2 and 5 mL) were performed to simulate thoracic duct embolization. For both glues, static polymerization times were longer when the iodized oil content was increased and when performed in high TG lymphatic fluid. In the dynamic experiments, the prolongation of polymerization due to the oil content and TG levels was less pronounced for both glue types. Increased lymphatic flow rates decreased embolization times for low glue/oil ratios while preventing embolization for high glue/oil ratios. Higher glucose flush volumes increased occlusion times. Polymerization times of acrylic glue in a lymphatic fluid are prolonged by increasing the iodized oil concentration and triglyceride concentration as well as by using larger volumes of glucose flush. Increased lymphatic flow rates decrease embolization times for low glue/oil ratios and may prevent embolization for high glue/oil ratios.

## 1. Introduction

Lymphatic embolization is increasingly employed in the treatment of patients with lymphatic/chylous effusions (e.g., chylothorax, chylous ascites or lymphoceles) [1]. In patients with thoracic chylous effusions, thoracic duct embolization (TDE) is already considered a primary treatment option [2,3].

Although there are some reports of the use of onyx in the lymphatic system [4], it is no longer recommended due to the porosity of the embolization cast, which may lead to interventional failure [5]. Therefore, most interventionalists performing lymphatic embolizations appear to use acrylic adhesives (cyanoacrylates, i.e., Truefill^®^; Histoacryl^®^; Glubran 2^®^) [6,7,8].

Cyanoacrylates are monomer glues that polymerize in the presence of anions and have clinically been used for embolization procedures as a mixture of glue and iodized oil for decades. However, their polymerization properties have so far mainly been investigated in blood for arterial embolization [9,10]. These data cannot easily be transferred to embolization in the lymphatic system as the lymphatic fluid composition and flow differ considerably from the arterial system [11].

Compared to arterial embolization, initial in vitro experiments demonstrated that polymerization of Histoacryl takes longer in lymphatic embolization and is influenced not only by the mixture ratio of glue to iodized oil but also by the triglyceride concentration of the lymphatic fluid [12]. Data on polymerization of other frequently employed acrylic adhesives within the lymphatic system, however, are lacking. Although the basic principles of acrylic adhesives of different vendors are similar, embolic properties may differ due to the different chemical compositions of the glues [13].

It also remains unclear whether procedural parameters, such as the lymph flow rate—which may vary in different patients—or the pre-embolization glucose flush volume have an impact on polymerization times in lymphatic embolizations [7]. Therefore, it was the goal of this study to investigate different acrylic adhesives for lymphatic interventions as well as the impact of lymph composition, lymph flow and the role of a pre-embolization glucose flush on polymerization in an in vitro setup.

## 2. Materials and Methods

Polymerization times were evaluated for Histoacryl [HA] (Braun, Melsungen, Germany) and Glubran II [GL] (GEM, Viareggio, Italy) mixed with iodized oil (Lipiodol, Villepinte, Guerbet, France) in 8 different ratios, ranging from 1:0 to 1:7 for two lymphatic fluid samples (lymph: low triglycerides (TG) < 50 mg/dl and chyle: high TG > 600 mg/dl), both in a static and a dynamic experimental setup as described previously [12]. Lymphatic fluid samples were taken from drained chylothoraces and underwent laboratory testing to investigate the concentration of triglycerides, total protein, leucocytes and sodium, potassium, calcium and chloride levels. The results of laboratory examinations of the two employed lymphatic fluid samples are listed in the online Supplement Table S1.

The institutional review board approved the anonymized use of the drained fluid samples for the in vitro experiments without the additional written informed consent of the patients.

### 2.1. Static Analysis of Polymerization Times

To determine the polymerization times of HA and GL mixed with iodized oil in lymphatic fluids, different mixtures were injected into the two lymphatic samples (one drop of the mixture per experiment). The polymerization experiments were subcategorized and defined as follows:

- A: HA in low TG;
- B: HA in high TG;
- C: GL in low TG;
- D: GL in high TG.

**Figure 1:**
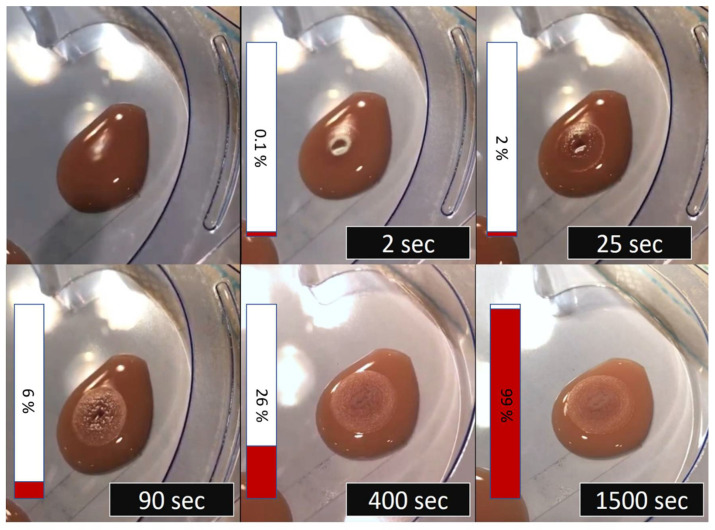
Example of typical static embolization testing setup. HA/iodized oil (in this case, with a ratio of 1:4) is dropped into a lymphatic sample (in this case, triglyceride content: 1111 mg/dL). The polymerization process is continuously monitored with a high temporal resolution camera (temporal resolution 0.07 s). The cessation of morphologic change marks the end of the experiment. The red and white bar on the left side of the images displays the percentage of total polymerization time.

The polymerization process was recorded by high temporal resolution video (60 frames/s) to document the morphologic changes in the polymer, as previously reported [14]. Polymerization was then evaluated by two radiologists. The completion of polymerization was defined as the time point when the morphologic changes ceased. All experiments were repeated five times. Figure 1 shows an example of a static embolization test.

### 2.2. Dynamic Analysis of Embolization Times

**Figure 2:**
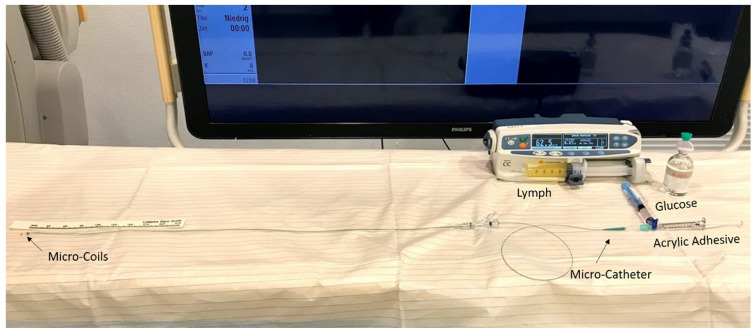
Basic testing setup for dynamic experiments: lymphatic fluid is continuously infused into a transfusion tube with microcoils placed within it. An acrylic adhesive mix with iodized oil is then applied into the tube via a microcatheter after flush-injection with 40% glucose. The catheter is then drawn back 10 cm while injecting embolic agent. The time until cessation of lymphatic flow (pressure increase > 60 mmHg) as well as the adhesive migration distance is measured.

A dynamic flow model was employed to simulate thoracic duct embolization. Dynamic polymerization times were investigated as previously reported [12]. A silicon transfusion tube with a diameter of 2.5 mm and a length of 150 cm was used to simulate a thoracic duct. As often performed in clinical TDE [3,7], microcoils (4 mm, VortX, Boston Scientific, Natick, MA, USA) were placed within the tube in a position 35 cm from its end in order to impede the lymphatic flow and to promote a mixture of the embolic agent with the surrounding fluid. After that, the tube was completely filled with the lymphatic fluid samples (low or high TG content) and a continuous flow was established by an injection pump. Tests were performed with two different lymphatic flow rates (62.5 mL/h and 125 mL/h). A microcatheter (Renegade, Boston Scientific, Natick, MA, USA), flushed with 40% glucose, was then inserted via a y-piece at the proximal end of the tube. After injection of 40% glucose (1, 2 or 5 mL), 3 mL of HA and GL mixed with iodized oil with different ratios were injected while the microcatheter was drawn back over a distance of 10 cm. The configuration of the resulting glue cast in the tube was recorded by video (60 frames/s). The time between the initial injection of acrylic adhesive/iodized oil and intraluminal pressure increased > 60 mmHg, leading to the cessation of flow as well as the distance of adhesive migration from the initial point of injection measured. All experiments were repeated three times. Figure 2 shows the setup of a dynamic embolization test.

### 2.3. Statistical Analysis

Statistical analyses were performed using commercially available statistical software (Prism version 8, GraphPad, La Jolla, CA, USA). Results are expressed as mean ± standard deviation (SD). For the intergroup (A, B, C and D) comparison of total polymerization times, a one-way analysis of variance (the Kruskal–Wallis test with Conover’s post hoc test) was employed for the various HA, GL/iodized oil mixtures. Moreover, *p*-values of < 0.05 were considered statistically significant.

## 3. Results

### 3.1. Static Polymerization

**Figure 3:**
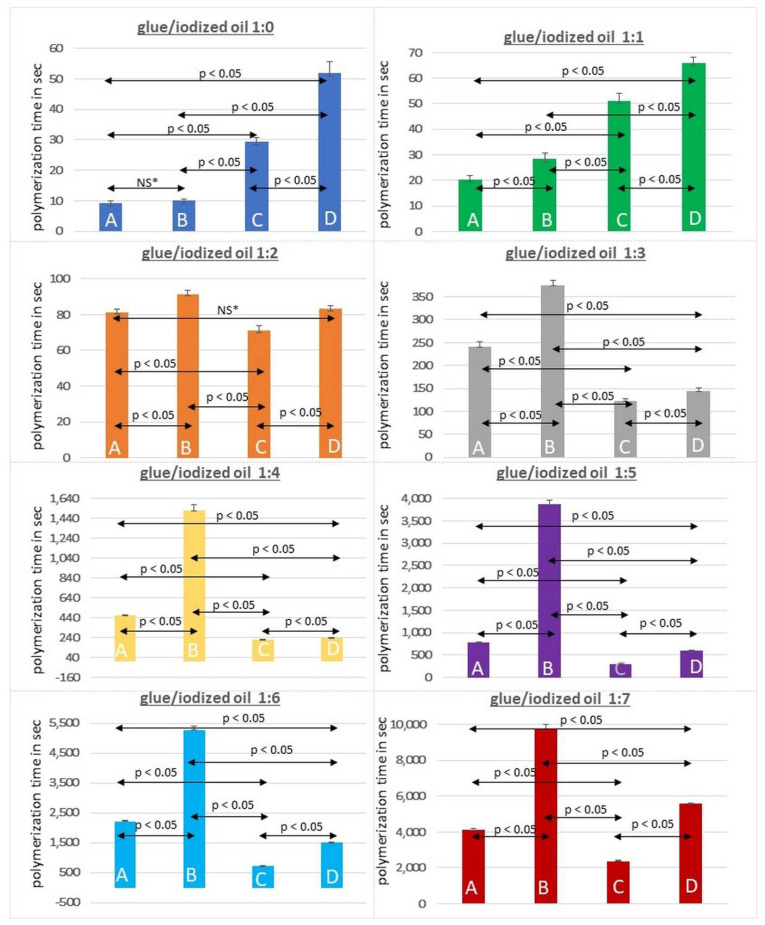
Polymerization times in seconds for HA (A & B) and GL (C & D) mixed with iodized oil (ratios 1:0 to 1:7) in low triglyceride fluid (low TG; < 50 mg/dl) (A & C) and high triglyceride fluid (high TG; > 600 mg/dL) (B & D). Arrows indicate results of intergroup comparison of total polymerization times using the Kruskal–Wallis test (one-way analysis of variance) and the Conover test (post hoc analysis). Polymerization times of pure HA (ratio 1:0), as well as high concentration HA (ratio 1:1), were significantly shorter than those of GL (Group A vs. C; Group B vs. D). For all other comparisons (ratios 1:2 to 1:7) GL showed significantly shorter polymerization times. NS* indicates non-significant results.

The results of the static evaluation (i.e., duration of total polymerization) are summarized in Figure 3 and listed in detail in the online Supplement Tables S2 and S3.

**Figure 4:**
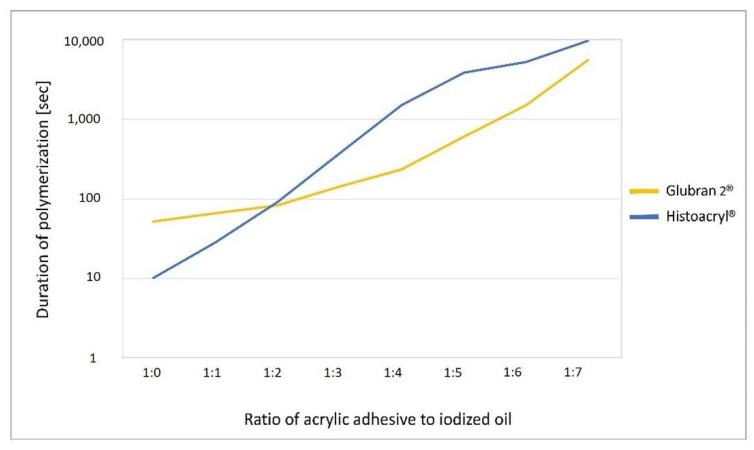
Duration of polymerization of acrylic adhesives (GL and HA) with varying ratios of glue/iodized oil (1:0 to 1:7) in high triglyceride (>600 mg/dL) fluid. Notice the logarithmic scale of polymerization duration. GL showed longer polymerization times than HA in tests with high glue concentration, while polymerization times of GL were shorter in tests with higher ratios of iodized oil.

The duration of static polymerization of both acrylic glues increased when higher ratios of iodized oil were employed (pure glue; ratio 1:0 in low TG fluid: 9.3 ± 0.6 s (HA) and 29.3 ± 1.5 (GL) vs. ratio 1:7: 4096.7 ± 110.6 s (HA) and 2363.3 ± 56.9 s (GL)). Furthermore, the duration of polymerization of both acrylic glues was also increased when embolization was performed in TG-rich lymphatic fluid (low vs. high TG with a ratio 1:3: 241 s vs. 374 s (HA) and 123 s vs. 143 s (GL); ratio 1:7: 4096 s vs. 9770 s (HA) and 2663 s vs. 5580 s (GL)). While GL showed longer polymerization times than HA in the tests with high glue concentrations (low TG: ratio 1:1: 20.3 ± 1.5 s (HA) vs. 51 ± 3 s (GL), high TG: ratio 1:1: 28.3 ± 2.5 s (HA) vs. 66 ± 2 s (GL)), polymerization times of GL were shorter than those of HA in the tests with higher ratios of iodized oil (low TG: ratio 1:4: 465.3 ± 4.5 s (HA) vs. 215.3 ± 5.5 s (GL), high TG: ratio 1:4: 1517 ± 60.8 s (HA) vs. 235.3 ± 5 s (GL). Figure 4 displays a comparison of the polymerization times of HA and GL in high TG fluids.

### 3.2. Dynamic Embolization

The results of the dynamic experiments are summarized in Table 1 (duration of dynamic embolization) and Table 2 (distance of dynamic embolization). The prolongation of polymerization due to the oil content and TG levels was less pronounced in the dynamic experiments than in the static experiments for both glue types. For low TG, total occlusion (pressure increase > 60 mmHg) of the silicon tube was observed in all cases for HA performed with a low lymph flow rate (62.5 mL/h) between 18.5 s (glucose flush 1 mL; ratio 1:1) and 289 s (glucose flush 5 mL; ratio 1:7), and for GL, between 16.5 s (glucose flush 1 mL; ratio 1:1) and 265 s (glucose flush 5 mL; ratio 1:7) with slightly faster occlusion times for GL. In the dynamic tests performed with high lymph flow rates (125 mL/h) total occlusion occurred quicker with a high concentration of acrylic adhesive for both HA (13 s; glucose flush 1 mL; ratio 1:1) and GL (12 s; glucose flush 1 mL; ratio 1:1). In tests with a low concentration of acrylic adhesive (ratio 1:7), glue casts dislocated past the coil package with both HA and GL (Table 1a).

For high TG levels, polymerization took considerably longer for both HA (35 s vs. 18.5 s; glucose flush 1 mL; ratio 1:1, flow rate 62.5 mL/h) and GL (26.5 s vs. 16.5; glucose flush 1 mL; ratio 1:1 flow rate 62.5 mL/h) (Table 1b). Glue casts dislocated past the coil package in tests with a low concentration of acrylic adhesive (ratio 1:7), both for the low and high flow rates.

An increase in the lymphatic flow rate (62.5 mL/h vs. 125 mL/h) leads to a reduction of embolization duration (slow flow, ratio 1:3, HA: 29.5–35.5 s, GL: 29–37.5 s; high flow, ratio 1:3, HA: 23–25 s, GL: 21–23.5 s; all *p* < 0.005) and to a slight increase in embolization distance (slow flow, ratio 1:3, HA: 21.5–32 cm, GL: 22.5–28 cm; high flow, ratio 1:3, HA: 29–29.5 cm, GL: 27–32 cm; all *p* < 0.05) (Table 2a + b). The impact of the glucose pre-flush volume was greater in low lymphatic flow tests (1 mL glucose, HA/GL ratio 1:1: 18.5/16.5 s; 5 mL glucose, HA/GL ratio 1:1: 24.5/20.5 s; all *p* < 0.05) than in high lymphatic flow tests (1 mL glucose, HA/GL ratio 1:1: 13/12 s; 5 ml glucose, HA/GL ratio 1:1: 15/14.5 s; all *p* < 0.05).

## 4. Discussion

Histoacryl^®^ (HA) is currently the most frequently employed embolic agent for lymphatic embolizations [5,6,7,15] and is the only embolic agent that has been systematically analyzed for this purpose [12]. As the number of lymphatic interventions is progressively increasing [3], the employment of other embolic agents (such as Glubran 2; GL) is expected to increase as well. While various studies have investigated the polymerization mechanisms and properties of HA in detail [16,17], data on GL is relatively sparse. Data on the polymerization properties of GL in the lymphatic system are lacking completely. What is known in general, is that GL, just like HA (as well as other acrylate adhesives, such as Trufill), is based on N-butyl-2-cyanoacrylate (NBCA). However, GL differs from other acrylate adhesives, as it is a mixture of two monomers: N-butyl-2-cyanoacrylate and metacryloxysulpholane (MS). MS, just like NBCA, is a cyanoacrylate monomer.

The polymerization of acrylate glues is initiated in one of three ways:

- Anionic polymerization initiation;
- Zwitterionic polymerization initiation;
- Radical polymerization initiation.

NBCA alone (as found in HA) primarily undergoes conventional anionically catalyzed polymerization [9]. In comparison, NBCA with added MS (as found in GL) mainly undergoes the polymerization pathway of radical polymerization initiation [18,19]. Adding MS to NBCA lowers the polymerization temperature from about 57 °C to 45 °C [10] and reduces the inflammatory effect. Furthermore, the addition of MS prevents the formation of bubbles and seems to lead to a more homogeneous and predictable polymerization of GL, which subsequently takes longer than that of HA [20,21]. However, apart from the studies conducted by Li et al. [13] and Levrier et al. [22], clinically applicable data regarding the polymerization properties of GL is currently sparse. As a result, the actual real-life polymerization properties of GL are not understood entirely [10].

The current results show that, comparable to published data in blood, the polymerization of GL in lymphatic fluids is slower than that of HA when using the glues without added iodized oil. This is most likely indebted to the addition of MS to the NBCA. However, both glues are only rarely used in their pure form for endovascular applications. Typically, iodized oil (Lipiodol) is added for embolization procedures. This is done for two reasons [7]: First, both glues are not radiopaque. The addition of Lipiodol helps to visualize glue propagation under fluoroscopy. Second, the polymerization of both glues would take place very fast, once exiting the catheter tip. Lipiodol lengthens the polymerization time and thus enables more controlled embolization.

When adding iodized oil to GL, the effect of MS seems to be inverted for mixture ratios above 1:2, and the polymerization duration becomes shorter for GL than for HA. It is of note that this phenomenon was also observed in non-lymphatic fluids [13]. A possible explanation may be that the polymerization of MS is less affected by iodized oil than NBCA.

As shown in a previous study, the polymerization duration of NBCA (±iodized oil) in lymph is not only dependent on the quantity of added iodized oil but also on the fatty content of the fluid embolization performed in [12]. The lipophilic/hydrophobic and non-ionic components of lymphatic/chylous fluids (especially triglycerides but also chylomicrons and fat-soluble vitamins [11]) also delay polymerization by shielding the NBCA from anions in the surroundings. This theory is supported by the current data, where the duration of HA and GL polymerization was directly dependent on the triglyceride concentrations of the samples in which polymerization was performed. This observation has clinical implications, especially in thoracic lymph vessels. The triglyceride concentration can vary considerably depending on the diet of the patient prior to embolization. Lymphatic leakages in the thorax typically are chylous when the patient is on a normal diet. Although the presence of chylomicrons is more specific for chylous fluid, triglyceride levels above 1.24 mmol/L (110 mg/dl) are accepted as a screening test for chylous fluid [23]. Especially as embolization may fail in triglyceride-rich lymphatic fluid when using a high dilution of the glue, the results support the recommendation to initiate a long-fatty acid-free diet prior to intervention. This lowers the triglyceride concentration as well as the flow rate of the lymphatic fluid within the thoracic duct [1].

Mean lymph flow in the thoracic duct—the largest lymph vessel in the human body—is approximately 1.3 mL per kg body weight per hour, amounting to around 2000 mL per day for a person of 70 kg body weight [24,25]. As stated above, clinical lymphatic interventions are typically performed following pre-interventional fasting/dietary modification, therefore lymphatic flow can be expected to be lower than 2000 mL per day [7,26]. In this respect, the employed flow rate for in vitro testing in the only study investigating the polymerization of HA in lymph published so far was higher than would physiologically be expected at 3600 mL per day [12]. In the present study, two different flow rates of 1500 mL and 3000 mL per day were therefore investigated to analyze the effects of low and high lymph flows on embolization. Interestingly, the results demonstrated that an increased lymphatic flow decreased the embolization duration, possibly due to a better mixing of lymphatic fluid and glue. However, the embolization distance increased with a higher lymph flow rate. When higher dilutions of the glues (ratios > 1:5) were used, this lead to an embolization distance longer than a typical thoracic duct (>40 cm) which clinically would carry the risk of venous migration of the glue. When increasing the dilution further to a ratio of 1:7, embolization may fail completely, especially at high lymph flow rates.

Apart from fatty meals, increased lymph production, e.g., due to liver, kidney, or heart disease can also lead to a significantly increased flow in the central lymphatic system that cannot be influenced other than by treatment of the underlying disease (e.g., by the creation of a transjugular intrahepatic portosystemic shunt (TIPS) in patients with liver cirrhosis) [27]. As our results have demonstrated, lymph flow has a significant impact on embolization distance; conditions associated with increased lymph flow should therefore be taken into account by the interventionalist when choosing the glue composition.

As clinically performed for arterial embolizations, glucose solution—as an anion-free fluid—is typically also injected for lymphatic embolizations via the microcatheter right before and after injection of cyanoacrylate to prevent blockage in the catheter and immediate polymerization at the tip of the catheter. However, in contrast to the arterial system, where glucose is immediately flushed away from the catheter tip after injection, in the lymphatic system, the flow is substantially lower. The injected glucose, therefore, remains around the catheter tip and may mitigate NBCA polymerization. For this hypothetical reason, large volume glucose injection prior to acrylic adhesive embolization has been advised against in lymphatic use [7]. However, whether the glucose flush volume has any effect on polymerization times has so far not been investigated. The results of the present study, for the first time, support this notion as a prolongation of polymerization times in dynamic experiments observed depending on the amount of glucose injected. Especially when injecting as much as 5 mL of glucose solution when using a high dilution of the glue, there was a considerable increase in polymerization durations and distance that may lead to venous spillage of glue when performed clinically. Therefore, small amounts of glucose—around 1 mL—seem to be preferable. However, no cases of complete embolization failure due to large glucose flush volumes were observed. This is most likely due to the fact that the effects of increased glucose volume and lymph flow rates partially counteract one another with smaller effects of glucose volume in higher lymph flow. However, although increased lymphatic flow (3000 mL/day) reduces the impact of glucose injection, embolization duration and distance are increased nonetheless, potentially causing interventional failure.

This in vitro study has several limitations. First, the experimental in vitro design limits the generalizability of the results to clinical applications. Static experiments were meant to evaluate the basic differences between HA and GL in lymph, and polymerization times cannot be transferred to a clinical situation in which fluid mixing takes place due to the flow. Second, dynamic experiments are also likely to yield systematically different results from clinical applications, as there will be an influence on the lymph vessel wall that cannot be simulated by a silicon tube. However, the results of the present study regarding HA are in agreement with a previous in vitro study and can serve as a point of orientation for interventionalists [12]. Third, dynamic experiments can further only be transferred to the thoracic duct or large lymph vessel embolization and not to interstitial lymph node embolization—a further interventional technique increasingly employed in the treatment of lymphatic leakage [3]. Fourth, we did not investigate all available acrylic embolic agents currently used for lymphatic embolization (i.e., Trufill). Trufill, like HA, is also a solely NBCA-based adhesive but differs from other adhesives. The Trufill kits also include separate ethiodized oil and tantalum powder. Unlike ethiodized oil, the tantalum powder included in the kit allows for better visualization without reducing viscosity or increasing the polymerization time. Generally, this feature is desirable in a high-flow milieu (such as AVMs) rather than in the lymphatic system. Otherwise, the properties of Trufill are expected to be equal to Histoacryl. Therefore, Trufill was not additionally examined. Fifth, the effect of different polymerization properties concerning homogeneity of the glue cast as well as the polymerization temperature differences between the glues cannot be investigated in the employed in vitro model and warrant further research. It remains unclear whether the lower polymerization temperature of Glubran II, for example, has any effect on the clinical results of lymphatic embolization.

## 5. Conclusions

The polymerization of acrylic glues depends not only on the ratio of iodized oil to adhesive but also on the triglyceride content of the lymphatic fluid in which embolization is performed as well as on the lymphatic flow rate and the amount of injected glucose. The polymerization properties of Histoacryl and Glubran II differ substantially depending on the employed adhesive-iodized oil ratio, with faster polymerization of Histoacryl in low ratios and faster polymerization of Glubran II in higher ratios. The results of this in vitro study indicate that careful adaptation of adhesive concentration should be performed for lymphatic embolization, depending on which acrylic adhesive is employed.

## Figures and Tables

**Table 1:** Embolization time.

(a) Dynamic Embolization Duration in Low TG Fluid.						
	Flow Rate 62.5 mL/h			Flow Rate 125 mL/h		
	Embolization Duration (Glucose 1 mL) [s]	Embolization Duration (Glucose 2 mL) [s]	Embolization Duration (Glucose 5 mL) [s]	Embolization Duration (Glucose 1 mL) [s]	Embolization Duration (Glucose 2 mL) [s]	Embolization Duration (Glucose 5 mL) [s]
HA 1:1	18.5 ± 1.2	19 ± 1.3	24.5 ± 1.3	13 ± 0.6	15.5 ± 1.4	15 ± 0.9
GL 1:1	16.5 ± 0.9	17.5 ± 1.9	20.5 ± 0.7	12 ± 1	12 ± 0.7	14.5 ± 1
HA 1:3	29.5 ± 1.2	31 ± 2.4	35.5 ± 1.9	23 ±1.9	26 ± 1.6	25 ± 1.6
GL 1:3	29 ± 1.1	27.5 ± 2.2	37.5 ± 1.8	21 ± 2.2	22 ± 1.3	23.5 ± 1.9
HA 1:5	41.5 ± 2.1	45 ± 3.4	52 ± 2.6	28.5 ± 1.9	27.5 ± 1.9	31.5 ± 2.3
GL 1:5	39.5 ± 1.7	43 ± 3.1	52.5 ± 2.5	27 ± 1.8	27 ± 1.8	30 ± 2.5
HA 1:7	243 ± 9.8	260 ± 11.7	289 ± 14.6	No occlusion	No occlusion	No occlusion
GL 1:7	189 ± 8.2	231 ± 10.8	265 ± 12.4	No occlusion	No occlusion	No occlusion
**(b) Dynamic Embolization Duration in High TG Fluid.**						
	Flow Rate 62.5 mL/h			Flow Rate 125 mL/h		
	Embolization Duration (Glucose 1 mL) [s]	Embolization Duration (Glucose 2 mL) [s]	Embolization Duration (Glucose 5 mL) [s]	Embolization Duration (Glucose 1 mL) [s]	Embolization Duration (Glucose 2 mL) [s]	Embolization Duration (Glucose 5 mL) [s]
HA 1:1	35 ± 1.4	38.5 ± 1.5	45.5 ± 2.1	23 ± 1.1	24 ± 1.3	36 ± 1.5
GL 1:1	26.5 ± 1.2	35 ± 1.3	41 ± 2.3	20 ± 0.8	19 ± 0.9	28 ± 1.4
HA 1:3	37.5 ± 2	41 ± 1.7	49.5 ± 2.6	30 ± 1.3	34 ± 1.6	41 ± 2.1
GL 1:3	30.5 ± 1.7	38 ± 1.8	40 ± 1.9	25 ± 1.4	25 ± 1.3	28 ± 1.1
HA 1:5	45.5 ± 2.1	49.5 ± 2.2	61 ± 3.2	36 ± 2.2	41 ± 1.7	44 ± 2.5
GL 1:5	40 ± 2.2	44 ± 2	54 ± 2.9	28 ± 1.8	32 ± 1.2	32 ± 1.6
HA 1:7	No occlusion	No occlusion	No occlusion	No occlusion	No occlusion	No occlusion
GL 1:7	No occlusion	No occlusion	No occlusion	No occlusion	No occlusion	No occlusion

(a) Dynamic embolization duration in low TG fluid. (b) Dynamic embolization duration in high TG fluid. Duration of embolization of acrylic adhesive/iodized oil in varying ratios as well as different volumes of glucose flush, leading to an intraluminal pressure increase > 60 mmHg. Histoacryl (HA) and Glubran II (GL) with varying ratios (1:1 to 1:7).

**Table 2:** Embolization distance.

(a) Dynamic Embolization Distance in Low TG Fluid.						
	Flow Rate 62.5 mL/h			Flow Rate 125 mL/h		
	Embolization Distance (Glucose 1 mL) [cm]	Embolization Distance (Glucose 2 mL) [cm]	Embolization Distance (Glucose 5 mL) [cm]	Embolization Distance (Glucose 1 mL) [cm]	Embolization Distance (Glucose 2 mL) [cm]	Embolization Distance (Glucose 5 mL) [cm]
HA 1:1	13.5 ± 1	15 ± 1	17.5 ± 1	16 ± 1	18.5 ± 2	18 ± 2
GL 1:1	10.5 ± 1	12.5 ± 1	16 ± 1	17 ± 1	17 ± 1	18.5 ± 1
HA 1:3	21.5 ± 2	26 ± 3	32 ± 2	29.5 ± 3	29 ± 2	29.5 ± 3
GL 1:3	22.5 ± 1	22.5 ± 2	28 ± 3	27 ± 2	27 ± 2	32 ± 2
HA 1:5	31.5 ± 2	36 ± 4	45 ± 4	36 ± 3	37 ± 4	41.5 ± 3
GL 1:5	28.5 ± 2	33.5 ± 4	40.5 ± 4	37.5 ± 3	42.5 ± 5	44 ± 3
HA 1:7	68 ± 4	74 ± 6	89 ± 7	No occlusion	No occlusion	No occlusion
GL 1:7	41 ± 2	58 ± 5	78 ± 5	No occlusion	No occlusion	No occlusion
**(b) Dynamic Embolization Distance in High TG Fluid.**						
	Flow Rate 62.5 mL/h			Flow Rate 125 mL/h		
	Embolization-Distance (Glucose 1 mL) [cm]	Embolization-Distance (Glucose 2 mL) [cm]	Embolization-Distance (Glucose 5 mL) [cm]	Embolization-Distance (Glucose 1 mL) [cm]	Embolization-Distance (Glucose 2 mL) [cm]	Embolization-Distance (Glucose 5 mL) [cm]
HA 1:1	20 ± 1	23 ± 2	26.5 ± 2	28 ± 2	26 ± 1	32 ± 2
GL 1:1	17 ± 1	17 ± 1	23 ± 1	24 ± 1	25 ± 2	28 ± 2
HA 1:3	24 ± 1	26 ± 2	32 ± 2	36 ± 2	38 ± 2	46 ± 3
GL 1:3	18,5 ± 1	26 ± 3	29 ± 2	31.5 ± 2	33.5 ± 2	36 ± 3
HA 1:5	38 ± 3	45.5 ± 3	52.5 ± 3	44 ± 3	49 ± 3	55 ± 4
GL 1:5	40.5 ± 3	41 ± 2	47 ± 3	42 ± 2	43 ± 3	45 ± 3
HA 1:7	No occlusion	No occlusion	No occlusion	No occlusion	No occlusion	No occlusion
GL 1:7	No occlusion	No occlusion	No occlusion	No occlusion	No occlusion	No occlusion

(a) Distance of how far the embolization cast migrated in low TG fluid from the microcatheter tip following injection of acrylic adhesive/iodized oil in varying ratios. Histoacryl (HA) and Glubran II (GL). (b) Distance of how far the embolization cast migrated in high TG fluid from the microcatheter tip following injection of acrylic adhesive/iodized oil in varying ratios. Histoacryl (HA) and Glubran II (GL).

## Data Availability

The data presented in this study are available on request from the corresponding author.

## Supplementary Materials

The following supporting information can be downloaded at: https://www.mdpi.com/article/10.3390/biomedicines10051195/s1, Table S1: Results of laboratory examinations of the employed lymphatic samples; Table S2: static embolization times; Table S3: Intergroup comparison static embolization.

## References

[1] Schild H.H., Strassburg C.P., Welz A., Kalff J. (2013). Treatment options in patients with chylothorax. Dtsch. Ärzteblatt Int..

[2] Schild H.H., Naehle C.P., Wilhelm K.E., Kuhl C.K., Thomas D., Meyer C., Textor J., Strunk H., Willinek W.A., Pieper C.C. (2015). Lymphatic Interventions for Treatment of Chylothorax. RöFo-Fortschr. Geb. Röntgenstrahlen Bildgeb. Verfahren..

[3] Pieper C.C., Hur S., Sommer C.M., Nadolski G., Maleux G., Kim J., Itkin M. (2019). Back to the Future: Lipiodol in Lymphography-From Diagnostics to Theranostics. Investig. Radiol..

[4] Nadolski G.J., Itkin M. (2018). Lymphangiography and thoracic duct embolization following unsuccessful thoracic duct ligation: Imaging findings and outcomes. J. Thorac. Cardiovasc. Surg..

[5] Itkin M., Chen E.H. (2011). Thoracic duct embolization. Semin. Interv. Radiol..

[6] Itkin M., Kucharczuk J.C., Kwak A., Trerotola S.O., Kaiser L.R. (2010). Nonoperative thoracic duct embolization for traumatic thoracic duct leak: Experience in 109 patients. J. Thorac. Cardiovasc. Surg..

[7] Chen E., Itkin M. (2011). Thoracic duct embolization for chylous leaks. Semin. Interv. Radiol..

[8] Lewis S.B., Srinivasa R.N., Shankar P.R., Bundy J.J., Gemmete J.J., Chick J.F.B. (2020). Thoracic Duct Embolization—Value Analysis Using a Time-Driven Activity-Based Costing Approach: A Single Institution Experience. Curr. Probl. Diagn. Radiol..

[9] Behan N., Birkinshaw C., Clarke N. (2001). Poly n-butyl cyanoacrylate nanoparticles: A mechanistic study of polymerisation and particle formation. Biomaterials.

[10] Li Y., Barthès-Biesel D., Salsac A.-V. (2017). Polymerization kinetics of n-butyl cyanoacrylate glues used for vascular embolization. J. Mech. Behav. Biomed. Mater..

[11] Merrigan B.A., Winter D.C., O’Sullivan G.C. (1997). Chylothorax. Br. J. Surg..

[12] Kuetting D., Schild H.H., Pieper C.C. (2019). In Vitro Evaluation of the Polymerization Properties of N-Butyl Cyanoacrylate/Iodized Oil Mixtures for Lymphatic Interventions. J. Vasc. Interv. Radiol..

[13] Li Y. (2017). In Vitro Characterization of Cyanoacrylate Embolic Glues Used for Vascular Embolization. Bachelor’s Thesis.

[14] Takasawa C., Seiji K., Matsunaga K., Matsuhashi T., Ohta M., Shida S., Takase K., Takahashi S. (2012). Properties of N-Butyl cyanoacrylate–Iodized oil mixtures for arterial embolization: In vitro and In vivo experiments. J. Vasc. Interv. Radiol..

[15] Schild H., Hirner A. (2001). Percutaneous translymphatic thoracic duct embolization for treatment of chylothorax. RöFo-Fortschr. Geb. Röntgenstrahlen Bildgeb. Verfahren..

[16] Donnelly E.F., Johnston D.S., Pepper D.C., Dunn D.J. (1977). Ionic and zwitterionic polymerization of n-alkyl 2-cyanoacrylates. J. Polym. Sci. Part C Polym. Lett..

[17] Limouzin C., Caviggia A., Ganachaud F., Hémery P. (2003). Anionic Polymerization of n-Butyl Cyanoacrylate in Emulsion and Miniemulsion. Macromolecules.

[18] Cho I. (2000). New ring-opening polymerizations for copolymers having controlled microstructures. Prog. Polym. Sci..

[19] García F., Garcia-Bernabé A., Compañ V., Díaz-Calleja R., Guzman J., Riande E. (2001). Relaxation Behavior of Acrylate and Methacrylate Polymers Containing Dioxacyclopentane Rings in the Side Chains. J. Polym. Sci. Part B Polym. Phys..

[20] Leonardi M., Barbara C., Simonetti L., Giardino R., Aldini N.N., Fini M., Martini L., Masetti L., Joechler M., Roncaroli F. (2002). Glubran 2: A new acrylic glue for neuroradiological endovascular use. Experimental study on animals. Interv. Neuroradiol. J. Peritherapeutic Neuroradiol. Surg. Proced. Relat. Neurosci..

[21] Leonardi M., Cenni P., Simonetti L., Bozzao A., Romano A., Bonamini M., Fantozzi L.M., Fini G. (2002). Glubran 2®:a new acrylic glue for neuroradiological endovascular use: A complementary histological study. Interv. Neuroradiol. J. Peritherapeutic Neuroradiol. Surg. Proced. Relat. Neurosci..

[22] Levrier O., Mekkaoui C., Rolland P.H., Murphy K., Cabrol P., Moulin G., Bartoli J.M., Raybaud C. (2003). Efficacy and low vascular toxicity of embolization with radical versus anionic polymerization of n-butyl-2-cyanoacrylate (NBCA). An experimental study in the swine. J. Neuroradiol..

[23] Staats B.A., Ellefson R.D., Budahn L.L., Dines D.E., Prakash U.B., Offord K. (1980). The lipoprotein profile of chylous and nonchylous pleural effusions. Mayo Clin. Proc..

[24] Hayashi S., Miyazaki M. (1999). Thoracic Duct: Visualization at Nonenhanced MR Lymphography—Initial Experience. Radiology.

[25] Hematti H., Mehran R.J. (2011). Anatomy of the Thoracic Duct. Thorac. Surg. Clin..

[26] Bierman H.R., Byron R.L., Kelly K.H., Gilfillan R.S., White L.P., Freeman N.E., Petrakis N.L., Singer G., Cordes F. (1953). The characteristics of thoracic duct lymph in man. J. Clin. Investig..

[27] Pieper C.C., Feißt A., Meyer C., Luetkens J., Praktiknjo M., Trebicka J., Attenberger U., Jansen C. (2021). Impact of transjugular intrahepatic portosystemic shunt creation on the central lymphatic system in liver cirrhosis. Sci. Rep..

