# Supplementary material for: In Vitro Evaluation of Acrylic Adhesives in Lymphatic Fluids-Influence of Glue Type and Procedural Parameters
Source: Biomedicines. 2022 May 21;10(5):1195. doi: 10.3390/biomedicines10051195 (PMC9138217; doi:10.3390/biomedicines10051195)
Supplement: Supplementary file 1 [file biomedicines-10-01195-s001.zip › biomedicines-1683291-SM-correct.pdf]

Table S1. Results of laboratory examinations of the employed lymphatic samples.

	Triglycerides (mg/dL)	Total Protein (g/L)	Leukocytes (/µL)	Erythrocytes (/µL)	Sodium (mmol/L)	Potassium (mmol/L)	Calcium (mmol/L)	Chloride (mmol/L)
**Lymphatic fluid**	13	21	579	< 1,000	147	4.25	1.61	111
**Chylous fluid**	1,111	7	3	1,000	146	4.5	1.25	113

Table S2. Static embolization times.

Glue/Lipiodol	Histoacryl Low TG (in sec) A	Histoacryl High TG (in sec) B	Glubran II Low TG (in sec) C	Glubran II High TG (in sec) D
**1:0**	9.3 ± 0.6	10.1 ± 0.5	29.3 ± 1.5	52 ± 3.6
**1:1**	20.3 ± 1.5	28.3 ± 2.5	51 ± 3	66 ± 2
**1:2**	81.3 ± 3.5	91.3 ± 3.5	71 ± 2	83 ± 4.6
**1:3**	241.3 ± 10	374 ± 12.5	123.3 ± 4.1	143.3 ± 7
**1:4**	465.3 ± 4.5	1517 ± 60.8	215.3 ± 5.5	235.3 ± 5
**1:5**	782 ± 7.5	3867 ± 100.7	302.7 ± 7.8	606 ± 8.2
**1:6**	2210.7 ± 49.1	5270 ± 140	718.3 ± 10.4	1506.7 ± 23
**1:7**	4096.7 ± 110.6	9770 ± 218	2363.3 ± 56.9	5580.3 ± 39.5

Polymerization times in seconds for Histoacryl (HA) and Glubran 2 (GL) mixed with iodized oil (ratios 1:0 to 1:7) in low triglyceride fluid (low TG; <50mg/dL) and high triglyceride fluid (high TG; >600 mg/dL).

Table S3. Intergroup comparison static embolization.

Ratio Glue/Iodized Oil	Kruskal Wallis Test	A vs. B	A vs. C	A vs. D	B vs. C	B vs. D	C vs. D
**1:0**	H(3) = 9.7. $p = 0.021$	ns	$p < 0.05$	$p < 0.05$	$p < 0.05$	$p < 0.05$	$p < 0.05$
**1:1**	H(3) = 10.4 $p = 0.0156$	$p < 0.05$	$p < 0.05$	$p < 0.05$	$p < 0.05$	$p < 0.05$	$p < 0.05$
**1:2**	H(3) = 9.2. $p = 0.027$	$p < 0.05$	$p < 0.05$	ns	$p < 0.05$	$p < 0.05$	$p < 0.05$
**1:3**	H(3) = 10.4. $p = 0.0156$	$p < 0.05$	$p < 0.05$	$p < 0.05$	$p < 0.05$	$p < 0.05$	$p < 0.05$
**1:4**	H(3) = 10.4. $p = 0.0156$	$p < 0.05$	$p < 0.05$	$p < 0.05$	$p < 0.05$	$p < 0.05$	$p < 0.05$
**1:5**	H(3) = 10.4. $p = 0.0156$	$p < 0.05$	$p < 0.05$	$p < 0.05$	$p < 0.05$	$p < 0.05$	$p < 0.05$
**1:6**	H(3) = 10.4. $p = 0.0156$	$p < 0.05$	$p < 0.05$	$p < 0.05$	$p < 0.05$	$p < 0.05$	$p < 0.05$
**1:7**	H(3) = 10.4. $p = 0.0156$	$p < 0.05$	$p < 0.05$	$p < 0.05$	$p < 0.05$	$p < 0.05$	$p < 0.05$

Inter-group comparison of total polymerization times for HA and GL in iodized oil using the Kruskal Wallis test (one way analysis of variance) and the Conover test (post-hoc analysis). A: Histoacryl in lymph; B: Histoacryl in chyle; C: Glubran II in lymph; D: Glubran II in chyle.
